# EMT and EGFR in CTCs cytokeratin negative non-metastatic breast cancer

**DOI:** 10.18632/oncotarget.2217

**Published:** 2014-07-14

**Authors:** Maria J. Serrano, Francisco G. Ortega, Maria J. Alvarez-Cubero, Rosa Nadal, Pedro Sanchez-Rovira, Marta Salido, María Rodríguez, Jose L. García-Puche, Miguel Delgado-Rodriguez, Francisco Solé, Maria A. García, Macarena Perán, Rafael Rosell, Juan A. Marchal, Jose A. Lorente

**Affiliations:** ^1^ GENYO. Pfizer-University of Granada-Andalusian Government Centre of Genomics and Oncology, Granada, Spain; ^2^ Laboratory of Genetic Identification-UGR, Department of Legal Medicine, University of Granada, Granada, Spain; ^3^ Hospital de Barcelona, Medical Oncology Department, Barcelona, Spain; ^4^ University of Jaén, Division of Preventive Medicine and Public Health, CIBERESP, Jaén, Spain; ^5^ Molecular Cytogenetics Laboratory; Pathology Department, Parc de Salut Mar-Hospital del Mar-IMIM-GRETNHE, Barcelona, Spain; ^6^ Medicine Department. Universitat Autònoma de Barcelona, Barcelona, Spain; ^7^ Institut de Recerca contra la Leucèmia Josep Carreras, Badalona, Spain; ^8^ Department of Oncology, Virgen de las Nieves, University Hospital, Granada, Spain; ^9^ Catalan Institute of Oncology, Hospital Germans Trias i Pujol, Badalona, Spain (RR); Pangaea Biotech SL, USP Dexeus University Institute, Barcelona, Spain (RR, MAM); ^10^ Biopathology and Regenerative Medicine Institute (IBIMER), Centre for Biomedical Research, University of Granada, Granada, Spain; ^11^ Department of Human Anatomy and Embryology, University of Granada, Granada, Spain

**Keywords:** Breast Cancer, Circulating Tumor Cells, EGFR, Epithelial-Mesenchymal Transition, Vimentin, Slug, Bcl-2, Apoptosis

## Abstract

Circulating tumor cells (CTCs) are frequently associated with epithelialmesenchymal transition (EMT). The objective of this study was to detect EMT phenotype through Vimentin (VIM) and Slug expression in cytokeratin (CK)-negative CTCs in non-metastatic breast cancer patients and to determine the importance of EGFR in the EMT phenomenon. In CK-negative CTCs samples, both VIM and Slug markers were co-expressed in the most of patients. Among patients EGFR+, half of them were positive for these EMT markers. Furthermore, after a systemic treatment 68% of patients switched from CK- to CK+ CTCs. In our experimental model we found that activation of EGFR signaling by its ligand on MCF-7 cells is sufficient to increase EMT phenotypes, to inhibit apoptotic events and to induce the loss of CK expression. The simultaneous detection of both EGFR and EMT markers in CTCs may improve prognostic or predictive information in patients with operable breast cancer.

## INTRODUCTION

Early detection and characterization of circulating tumor cells (CTCs) have a prominent role as a prognostic and predictive factor in several types of solid tumors [1] and, especially in breast cancer (BC) evolution. Nevertheless, our knowledge of the biological properties of CTCs is still limited, and it is broadly accepted that during tumorigenesis CTCs acquire features of invasiveness and motility, surviving in hostile environments, such as the bloodstream [2,3]. These phenotypic changes are associated with or are at least partially a consequence of the epithelial-mesenchymal transition (EMT) phenomenon [4]. The implication of EMT in BC has been established. In fact, CTCs of primary tumors express EMT markers and the presence of mesenchymal markers is higher in metastatic BC patients than in early stage BC patients, suggesting that EMT phenotype is directly related to the metastatic potential of CTCs.

The process of EMT involves the formation of metastatic cancer cells that gain the expression of mesenchymal markers such as vimentin (VIM) or Slug and the loss of epithelial markers including the epithelial cell adhesion molecule (EpCAM) or cytokeratin (CK), acquiring a mesenchymal or semi-mesenchymal phenotype [5,6]. Furthermore, the presence of this EMT phenotype has been associated not only with the metastatic potential of CTCs but also with the capacity of these CTCs to present drug resistance [7]. In fact, high levels of VIM expression in cancer patients are correlated with a poor prognosis [8] and, the simultaneous expression of VIM and CK in BC cells seems to be associated with lower survival rates in BC patients. Also, Slug has been associated with low life expectancy in a variety of human cancers [9].

Moreover, EGFR signaling is important in normal epithelial development and in tumor cell proliferation, motility, survival and metastasis and it is known to be overexpressed in BCs, especially in triple negative BC cases [10]. Interestingly, it has been demonstrated that EGFR inhibition suppress EMT and consequently decreases cell migration and invasion ability [11].

To the best of our knowledge there are no studies that explore the relationship between EGFR expression and EMT markers (VIM and Slug) in CTCs isolated from BC patients. Since EGFR may promote proliferation and progression in BC and VIM and Slug are potential regulators of cell adhesion and migration, our study aimed to determine the correlation between the expression of these EMT markers and the up-regulation of EGFR expression in CTCs negatives for CK. Finally, we studied if CK-CTCs with EMT features could be used as indicators to evaluate the prognosis of operable BC patients.

## RESULTS

### Correlation of VIM and Slug expression in CTCs with clinical and pathological characteristics

Patients included in this study were consistent with an unselected early and locally advanced BC population. Clinical-pathological characteristics were stratified according to the baseline VIM and Slug expression in CK-negative CTCs status (Table 1). Clinical tumor size was the only primary tumor features that correlated with VIM^+^ and Slug^+^ CTCs (*p*= 0.048 and *p*= 0.047, respectively). No significant correlation was found between VIM+/Slug+ expression in CTCs and the clinical characteristics of patients, including age (<50 vs ≥50 years old), histology, nodal status (N0 vs N1-2), grade (gI vs gII vs gIII), hormonal status (RH^+^ vs RH^−^), p53 status (positive vs negative) or KI67 (<14 vs≥14). There was no correlation between VIM or Slug/CK-negative CTC status and BC subtypes (luminal vs triple negative/HER2 amplified) (data not shown).

**Table 1:** Clinical-pathological characteristics according to the baseline status

		N(%) VIM+	N(%) VIM-	p(χ^2^)	N(%) SLUG+	N(%) SLUG-	p(χ^2^)
Age	≤ 50 > 50	8 (33.33) 10 (23.26)	16 (66.67) 33 (76.74)	0.270	8 (30.77) 9 (20)	18 (69.23) 36 (80)	0.305
Histology	Ductal Others	17 (29.31) 1(11.11)	41 (70.69) 8 (88.89)	0.258	16 (25.81) 1 (11.11)	46 (74.19) 8 (88.89)	0.309
Clinical Tumor Size	≤ 2cm > 2-5cm > 5cm	10 (31.25) 3 (12) 5 (50)	22 (68.75) 2 (88) 5 (50)	0.048*	10 (31.25) 3 (10.34) 4 (40)	22 (68.75) 26 (89.66) 6 (60)	0.047*
Clinical Node Status	N0 N +	17 (29.82) 1 (10)	40 (70.18) 9 (90)	0.182	16 (26.67) 1 (9.09)	44 (73.33) 10 (90.91)	0.196
Grade	I II III	2(13.33) 10 (37.04) 4(20)	13 (86.67) 17 (62.96) 16 (80)	0.224	2 (13.33) 9 (32.14) 4 (17.39)	13 (86.67) 19 (67.86) 19 (82.61)	0.361
Hormonal Status	RH- RH +	3 (21.43) 15 (28.30)	11 (78.57) 38 (71.70)	0.442	3 (20) 14 (25)	12 (80) 42 (75)	0.490
HER2 Status	HER2 - HER2 +	16 (28.7) 2 (20)	41 (71.93) 8 (80)	0.460	16 (26.23) 1 (10)	45 (73.77) 9 (90)	0.248
P53 Status	P53 - P53 +	10 (27.03) 3(30)	27 (72.97) 7(70)	0.569	9 (23.68) 3 (30)	29 (76.32) 7 (70)	0.483
Ki67%	(KI67-)< 14% (KI67+)≥ 14%	7 (28) 11 (26.19)	18 (72) 31 (73.81)	0.544	7 (28) 10 (21.14)	18 (72) 36 (78.26)	0.377

### Correlation of VIM and Slug expression in CK-negative CTCs with EGFR, CD133, TOPO2/HER2 biomarkers

**Figure 1:**
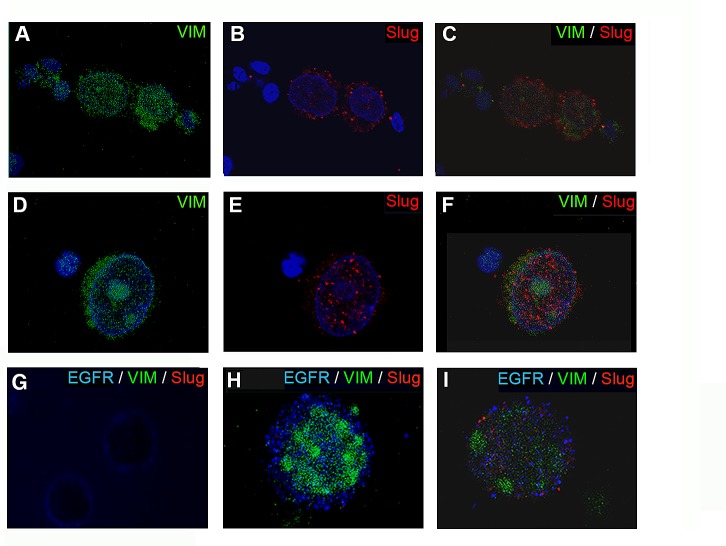
Epifluorescence microscopy images of CTCs stained for VIM and Slug expression (A-F) CTCs CK-negative/VIM^+^/Slug^+^ from BC patients stained in green for vimentin and red for Slug. Nuclei were stained in blue (DAPI). Representative images showing of a different cellular distribution of Slug, (B) cytoplasmic or (E) nuclear. (G-I) VIM and Slug expression in EGFR positive cells. (G) SKBR cells used as positive control for EGFR (blue) and negative for VIM (green) and Slug (red). (H-I) Representative images of the different phenotypes found in CK-negative/EGFR^+^CTCs from BC patients. (H) CTC CK-negative/EGFR^+^/VIM^+^/Slug^−^ sample. (I) CTC CK-negative/EGFR^+^/VIM^+^/Slug^+^ sample. Note: nuclei of the cells were stained with DAPI after the visualization of VIM, EGFR and Slug expression. Original magnification 40x (A-C, G); 63 x (D-F); and 100x (H and I).

To identify EMT-like CTCs, 78 blood samples multi-CK-negative were stained with antibodies specific to CD45 (a leukocyte marker), VIM and Slug as described in material and method (Supplementary Fig. 1S). Samples with high background staining were discarded. 67 samples were considered for VIM analysis. From these samples, 18 (26.9%) showed VIM expression and 49 (73.1%) were negative. 71 samples were analyzed for Slug expression. 17 (23.9%) of these patients were positive for Slug expression and 54 (76.1%) were negative. Interestingly, when Slug was positive in CK-negative CTCs, the VIM marker was co-expressed in 94.4% (17/18) of the cells. However, VIM expression was not always co-expressed with Slug (1/18; 5.56%). Thus, VIM negative CTCs were also negative for Slug (49; 100%). In addition, we found that Slug was mainly detected in the cytoplasm (Fig. 1B); although some samples showed a nuclear staining pattern as well (Fig. 1E).

Additionally, in order to examine the correlation of EFGR, TOPO2/HER2 and CD133 with VIM and Slug expression, we studied the presence of these biomarkers in patients with CK-negative CTCs before any treatment (Table 2). 67 patients were analyzed for VIM and EGFR expression. In samples EGFR negative we found that 9 (18.4%) were VIM^+^ and 40 (81.6%) were VIM^−^. In EGFR positive samples, 10 (55.5%) were VIM^−^ and 8 (44.5%) were VIM^+^ (*p*= 0.044). In addition, EGFR expression was analyzed in 71 patients positive for Slug marker. 9 (17.3%) samples were EGFR^−^/Slug^+^, 43 (82.7%) were EGFR^−^/Slug^−^, 11 (57.9%) were EGFR^+^/Slug^−^ and 8 (42.1%) were EGFR^+^/Slug^+^ (*p*= 0.030). A representative picture of VIM and Slug expression in EGFR positive cells is shown in Fig. 1D and E. We did not find any correlation between CD133 expression and EMT markers in CK-negative CTCs. Moreover, TOPO2/HER2 amplification was detected in only 8 of the 67 patients and only 3 samples were VIM^+^ (37.5%; *p*= 0.366). Slug expression was detected in 3 of the 12 (25%) TOPO2/HER2^+^ patients (*p*= 0.592) (Table 3).

**Table 2:** Correlation between EMT markers (VIM and Slug) and EGFR, CD133 and TOPO2/HER2 in CK-negative CTCs

	EMT MARKERS	VIM+ N (%)	VIM- N (%)	*p*	SLUG+ N (%)	SLUG- N (%)	*p*
EGFR	EGFR+ EGFR-	8 (44.5) 9 (18.4)	10 (55.5) 40 (81.6)	0.044	8 (42.1) 9 (17.3)	11 (57.9) 43 (82.7)	0.030
CD133	CD133+ CD133-	0 (0) 18 (26.9)	0 (0) 49 (73.1)	-	0 (0) 17 (24.0)	0 (0) 54 (76)	-
TOPO2/HER2	TOPO2/HER2+ TOPO2/HER2-	3 (37.5) 15 (25.4)	5 (62.5) 44 (74.6)	0.366	3 (25) 14 (23.7)	9 (75) 45 (763)	0.592

**Table 3:** Relationship between EGFR expression and recurrence in BC patients negative for CK expression after systemic treatment

CTC-EGFR	No Recurrence	Recurrence	*p*
CTC-CK-/EGFR- CTC-CK-/EGFR+	21 (95.45) 1 (33.33)	1 (4.55) 2 (66.66)	0.032

### Modifications in CK, EGFR and apoptotic markers after induction of EMT phenomenon in MCF-7 cells

**Figure 2:**
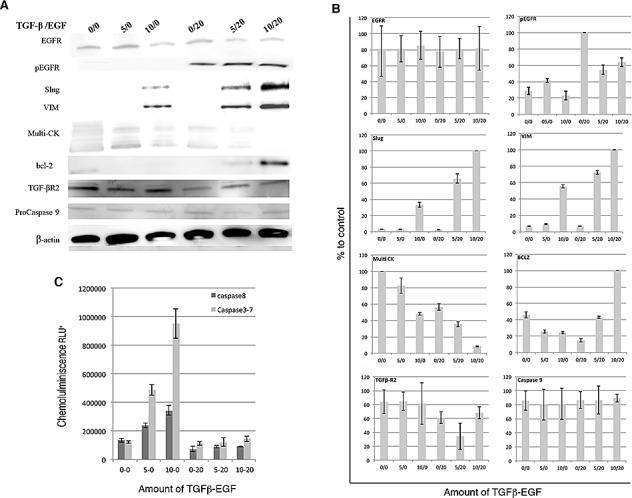
Effects of TGFβ1 and EGF on EMT, apoptosis and CK expression in MCF-7 cells MCF-7 cells were treated with TGFβ1 (0.5 and 10 ng/ml), EGF (20 ng/ml), or a combination of TGFβ1 and EGF (5 or 10ng/ml and 20ng/ml, respectively) for 72 hours. (A) Western blots analysis using anti-EGFR, anti-phospho EGFR, anti-Slug, anti-VIM, anti-Bcl-2, anti-caspase 9, anti-multi-CK, anti-TGFβ R2 anti-β-actin antibodies. β-actin was used as loading control. (B) Densitometric analysis related to control. (C) Luminescence detection of Caspase-3, -7 and -8 activities. Enzymatic activity was expressed in relative light units (RLU). Data represent the mean ± SD from three independent experiments (*p<0.05, ***p<0.01 vs. control).

To further explain the relationship between EGFR expression in CK-negative CTCs and EMT process we developed an experimental model using MCF-7 tumor cells. MCF-7 cells were stimulated with TGFβ_1_ and/or EGF at several concentrations for 72 h. Firstly, we analyzed by western blotting the switch in the pattern of expression of VIM, Slug, Pan-CK and EGFR status and changes in the apoptosis mediators Bcl-2 and pro-caspase 9 (Fig. 2). Our results showed that the induction with TGFβ_1_/EGF combination maintained EGFR activation, which was accompanied by a significant enhanced expression of both VIM and Slug markers and the complete inhibition in Pan-CK expression. Moreover, TGFβ_1_/EGF combined treatment involved a significant induction in Bcl-2 expression and no modifications in pro-caspase-9. Furthermore, caspase-3, -7 and -8 activities increased after induction with 10 nM TGFβ_1_ and significantly decreased when EGF was added (Fig. 2C). The significant Bcl-2 and VIM and Slug induction together the caspase 3/7 and 8 decreasing demonstrate the inhibition of extrinsic apoptotic pathway after acquisition of EMT phenotype in EGFR^+^/CK-negative cells.

**Figure 3:**
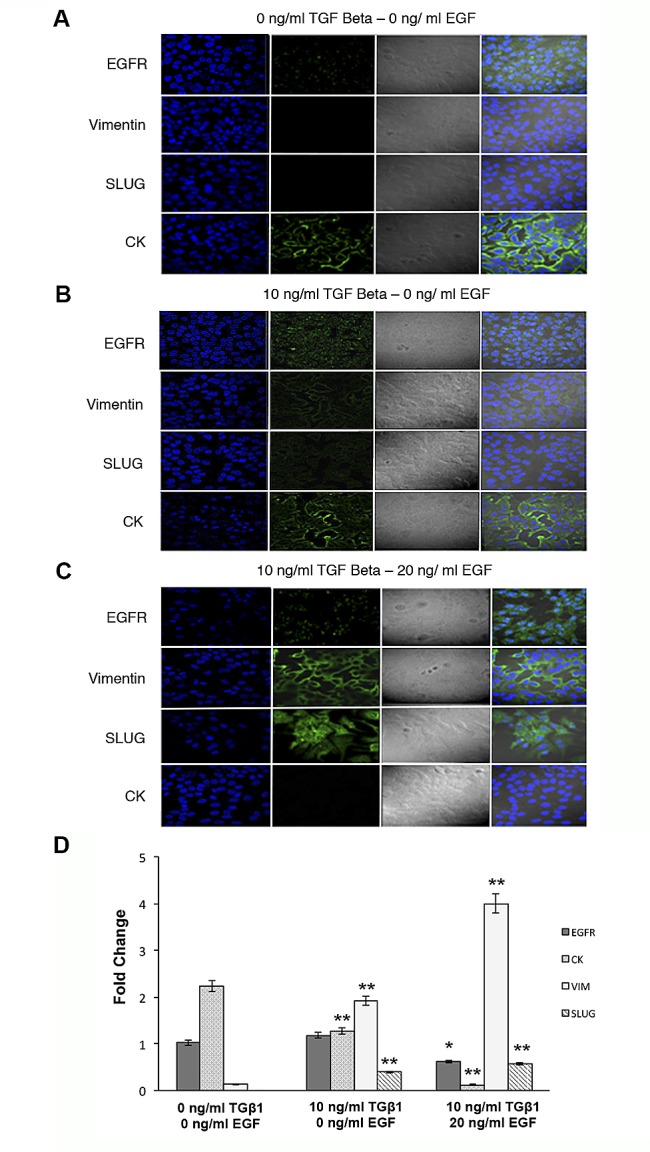
Expression of EGFR, VIM, Slug and CK in MCF-7 cells after TGFβ1 and/or EGF induction Confocal microscopy and RT-qPCR examination of MCF-7 cells treated with TGFβ1 (0 and 10 ng/ml), EGF (20 ng/ml), or a combination of TGFβ1 and EGF (10 ng/ml and 20 ng/ml, respectively) for 72 h. Representative immunofluorescence and light microscopic images of EGFR, VIM, Slug and CK staining. Microscope fields shown are representative of at least three different assessments. (A) Non-treated control cells showed marked staining for CK and a low EGFR expression. (B) TGFβ1-treated cells displayed the expression of VIM and Slug and a decrease in CK staining. (C) TGFβ1/EGF combined treatment induced a high expression of EGFR, VIM and Slug and the total disappearance of CK. Nuclei are stained with DAPI (blue). Original magnification 20x. (D) RT-qPCR analysis of mRNA *EGFR, CK8, Slug* and *VIM* expression levels. Data represent the mean ± SD from three independent experiments (*p<0.05, ***p<0.01 vs. control non-treated cells).

Finally, immunofluorescence analysis confirmed data obtained by western blot. The morphologic changes of MCF-7 cells after transformation with TGFβ_1_ and EGF included loss of cell adhesion, reduced cell-cell contact, and increased pseudopodia. Moreover, confocal microscopy studies showed an increased and robust expression of VIM and Slug, which was located in both nucleus and cytoplasm and the total disappearance of CK staining after TGFβ_1_/EGF combined treatment (Fig.3 A-C). This increase was also detected by RT-qPCR technique, where the relative mRNA levels of*VIM* and *Slug* significantly enhanced whereas *CK8* expression practically disappeared after combined treatment. *EGFR* expression decreased only after TGFβ_1_/EGF combined treatment suggesting that changes observed in the expression of protein level should be due to post-transcriptional modifications (Fig. 3D). These results confirm the loss of epithelial phenotype and the acquisition of mesenchymal markers in CK negative/EGFR^+^ MCF7 cells (Fig. 3).

### Treatment with TGFβ1 or TGFβ1/EGF promotes cell motility

**Figure 4:**
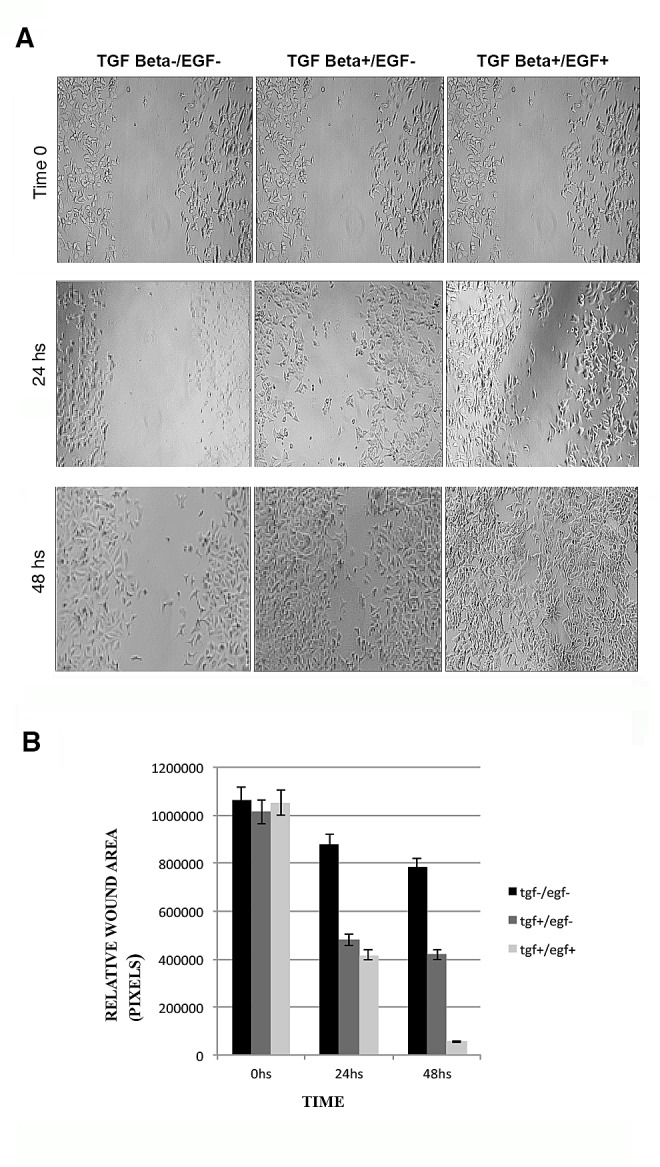
Wound healing assays in MCF-7 cells after induction with TGFβ1 and/or EGF (A) Migration of the cells to the wound was visualized at 0, 24 and 48 h with an inverted phase-contrast microscope. Original magnification 10x. (B) Quantification of cell migration was done by counting the free pixels inside and outside of the detection zone (Data were analyzed as percentages of the control cells in three independent experiments. *p < 0.05 and **p < 0.01 were considered significant).

Since EMT is generally associated with a migratory phenotype that is indispensable for cancer metastasis, we performed wound-healing assays in MCF-7 cells treated with TGFβ_1_ or TGFβ_1_/EGF. Our purpose was to demonstrate that continued activation of EGFR and the corresponding CK inhibition is associated with a higher migratory potential. Our results showed that TGFβ_1_ or TGFβ_1_/EGF exposition significantly increased the motility in MCF7 cells in a time-dependant manner with respect to control non-induced cells (Fig. 4A). This enhanced migration was more evident in the combined treatment in comparison with the TGFβ_1_ induction alone (Fig. 4B).

### EGFR and BC recurrence after systemic treatment

78 patients classified as CK negative received systemic treatment. At the end of treatment it was notable that only 25 patients have CK-negative CTCs and, thus, 53 switched from CK negative to CK positive CTCs. 22 cases of the 25 CK negative CTCs were EGFR negative and only one (4.5%) patient showed cancer recurrence. Interestingly, two patients (66.66%) EGFR^+^ CK negative presented relapse of the disease (*p*=0.032) (Table 3).

## DISCUSSION

The systemic nature of BC is defined by the dissemination of early tumor cells, even with relatively small tumors. In this scenario, the metastatic process involves the dissemination of CTCs by the blood and the lymphatic system prior to the colonization of distant organs. Several studies have observed the presence of CTCs in peripheral blood as the precursor of clinically manifest distant metastases [12].

The metastatic process is comprised of phenotypic alterations that are mediated by genetic changes. Among these phenotypic variations the EMT process modulates cell survival, migration and resistance to anoikis and apoptosis [13]. Epithelial cells undergoing EMT lose their characteristics and acquire a mesenchymal phenotype, which include loss of epithelial markers (E-cadherin and CK) and gain of mesenchymal markers (N-cadherin and VIM) [14].

Currently, the majority of techniques used to isolate and detect CTCs are based on the expression of epithelial markers. Consequently, if the EMT process is essential to the migration and survival of tumor cells before the colonization of target organs, then, an important aggressive subpopulation of tumor cells may not be detected. Recently, it has been shown that patients with poor prognosis factors had CTCs undetectable by routinely used techniques.

In this study, we evaluated the expression of EMT markers in a group of primary BC patients, who were negative for CK in the basal analysis. Moreover, we further explored if EGFR expression in these patients was correlated with the acquisition of the EMT phenotype. To test our hypothesis that an important cell tumor fraction is not detected and, therefore, we can obtain false-negative samples, we analyzed VIM and Slug in CK-negative CTCs in BC patients. Approximately, 27 percent of samples negative for CK expression were positive for VIM and 24 percent for Slug, respectively. Similar percentages have been obtained by Raimondi's group, which showed that 34% of CK negative patients were VIM and fibronectin positive [15]. In addition, our results showed that in most of the CK-negative CTCs, Slug and VIM were co-expressed. An “*in vivo*” model with transplantable human breast tumor cells uniquely capable of spontaneous EMT events was used to demonstrate that in primary xenograft tissues Slug was overexpressed in VIM+ areas [16]. These results indicate the need to optimize CTCs isolation and detection methods including the surface EMT markers. New methods based in isolating by size may be a powerful tool to detect different CTC subpopulations [17].

Interestingly, we found a direct relationship between tumor size and VIM and Slug expression in CK-negative CTCs. In fact, it has been demonstrated that EMT progression is responsible for increased tumor growth and metastasis in prostate and BC xenograft experiments [18]. Moreover, in prostate carcinoma the increase of the E-cadherin epithelial marker was inversely correlated with size of the metastasis and this expression was increased compared to the primary lesion [19]. Our results suggest the important role of VIM/Slug EMT markers in tumor growth of primary BC.

Our data show that Slug was mainly distributed in the cytoplasm of the cells, although nuclear staining was also detected. A recent study demonstrated that cytoplasmic Slug induces invasive finger-like protrusions termed invadopodia in pancreatic tumors cells by intracellular F-actin polymerization. This process of modulation of the cytoskeletal structure is directly related to a higher invasive and metastatic capacity [20].

Several authors have correlated EGFR expression with a poor prognosis in BC patients. It has also been demonstrated that the EGFR pathway controls several important biological processes, including cellular proliferation, angiogenesis and inhibition of apoptosis [21]. The EMT program is associated with cellular pathways that confer new characteristics to the cells, such as apoptosis resistance, migration capacity and chemo and radioresistance [22]. We found a statistically significant correlation between EGFR^+^ CTCs and CK^−^/VIM^+^/Slug^+^ CTCs in approximately 40% of patients. In the same context, a recent study showed that HER2 overexpression in BC cells is accompanied by partial EMT-like transition through the activation of Wnt/β-catenin signaling pathway leading to transactivation of EGFR and promoting EMT-like transition [23]. Also, Lo et al. examined the role of the EGFR pathway in tumor cell lines and histological types of primary BC patients. In this study, they reported that high EGFR expression induced EMT, with subsequent Twist, Snail and Slug induction [24]. However, no clinical studies have been done to study the relationship between EGFR status, EMT phenotype and CTCs in non-metastatic BC, due to the extremely difficult to characterize CTCs during the EMT process. Our results suggest that CK-negative CTCs with high EGFR expression induced EMT, and this phenotypic transition could involve the EGFR-mediated activation of VIM and the subsequent VIM-activated Slug gene expression.

To strengthen our hypothesis, we determined the role of EGFR in mediating EMT and CK expression on the MCF7 tumor cell line that possesses epithelial characteristics. MCF7 was stimulated with TGFβ_1_, a potent initiator of mesenchymal transformation [4], or TGFβ_1_/EGF to induce EMT phenotype. We found that the activation of EGFR up-regulated VIM and Slug mesenchymal markers and down-regulated pan-CK epithelial markers. Our results suggest that the activation of EGFR signaling by its ligand and the presence of TGFβ_1_ induces EMT and subsequently inhibits CK expression (Supplementary Fig. 2S). Recently, it has been demonstrated that CTCs from patients with metastatic BC had predominantly mesenchymal phenotypes [25] and that EGF can induce EMT-like effects including the up-regulation of Twist through the EGFR pathway [24], which agree with our experimental data. Interestingly, we also detected that cells treated with TGFβ_1_ alone showed a dose-dependent increment in caspase 3/7 and 8 activity on the contrary, TGFβ_1_/EGF combination overexpressed Bcl-2 and significantly decreased caspases activity in MCF-7/EMT ^+^/CK-negative cells. TGFβ_1_–induced apoptosis has been considered to be largely dependent on caspase activation [26]. In epithelial cells, TGFβ_1_ is able to induce both cell apoptosis and EMT in the same cell type, in a cell cycle-related manner, in which apoptosis took place at G_2_/M phase and EMT in G_1_/S phase. Moreover, the EGFR activation in TGFβ_1_ treated cells was sufficient to increase EMT phenotypes, to inhibit apoptotic events and to induce loss of CK expression. Down-regulation of epithelial cell marker expression occurs concomitantly with, and as a driver of, a wound-healing response and due to loss of cell to cell contact between cell-basement membranes and their structural/functional polarity [27]. Signaling through the EGF receptor is known to influence the apoptotic resistance and the invasive potential of certain cancers, and this may be associated with the adoption of a transdifferentiated phenotype. In human renal cells, it has been demonstrated that EGF promotes EMT by offsetting the pro-apoptotic effects of TGFβ_1_ without preventing its EMT-inducing effect, thereby facilitating the improved survival of cells undergoing EMT [27], in concordance with our results of EGFR activated human BC cells. Moreover, in squamous carcinoma both the over expression of Bcl-2 or EGFR activation induced EMT, promoting cell migration and invasion via the ERK1/2 and PI3K-regulated MMP-9/E-cadherin signaling pathways [28]. These experimental data could explain the recurrence related to CK negative/EGFR^+^CTCs of non-metastatic BC patients. However, further studies with a higher number of patients are needed to confirm this hypothesis.

On the other hand, in a recent study, Chang et al. [29] found a correlation between the development of resistance to gefitinib and Slug expression in non-small cell lung cancer patients with EGFR mutation. Our preliminary results showed that patients with CK-negative CTCs that expressed EGFR showed poor progression free survival (PFS) after receiving systemic treatment. Although we did not correlate VIM and Slug expression with PFS these results suggest that EGFR expression in CTCs is necessary for the progression of the disease and that EMT markers may contribute to the survival of CTCs in peripheral blood. Nevertheless, the number of patients is too low to really give any clear conclusion and it opens a possibility for studies implicating higher number of patients.

However, the complexity of the dissemination process still needs further clarifications since a heterogeneous population of CTCs is believed to spread into the circulation. In fact, we found CTCs with three different phenotypes: i) CK^+^/EGFR^+^/Vim^−^/Slug^−^; ii) CK^−^/EGFR^+^/Vim^−^/Slug^−^; and iii) CK^−^/EGFR^+^/Vim^−^/Slug^+^. Moreover, we found that patients CK^−^/EMT+ at baselines were CK^+^ after treatment. Actually, it has been shown that after targeted therapy, CTCs from responding BC patients were fewer in number and presented more characteristic of an epithelial phenotype than CTCs from refractory patients, which were more numerous and retained or acquired a mesenchymal phenotype [25]. In these patients with metastatic BC patients were detected a significant number of CTCs exhibiting a partial or a full-blown EMT phenotype, supportive of an EMT-driven mechanism. Interestingly, a large fraction of the CTCs were either double epithelial/mesenchymal or mesenchymal-positive, particularly among the HER2^+^ and triple negative subtypes [25]. These findings could be explained by the transient and reversible nature of EMT processes in CTCs since an epithelial phenotype is favorable in the latter stages of the metastatic cascade and in the metastasis growth. EMT occurs in primary tumors, providing the cells with an enhanced ability to intravasate and generates CTCs. On the other hand, MET phenomena would occur to favor the metastatic growth in secondary organs, emphasizing the dynamic plasticity of the process [16,19]. It is important to highlight that our study was focused in patients that presented a negative expression of CK. We show that is necessary to optimize CTC detection methods by including EMT markers able to distinguish between diverse phenotypes that could be linked to tumor aggressiveness.

Detecting changes in cell phenotype could be the key for developing new therapeutic targets or more effective therapeutic regimens. Furthermore, present results may indicate that different signaling pathways are activated in CTCs to promote EMT, leading not only to the possibility of progression but also survival in a hostile microenvironment. Our findings suggest that the simultaneous detection of EGFR, EMT antigens (VIM and Slug) and CK in CTCs by enrichment methods may contribute to the better detection of CTC subpopulations and improve prognostic or predictive information during systemic therapy in patients with operable BC.

## MATERIALS AND METHODS

### Patients

78 stage I to IIIC BC patients were enrolled from the Breast Cancer Unit of the University Hospital of Jaén and Hospital del Mar of Barcelona from March 2009 to September 2010. The inclusion criteria were the histological diagnosis of BC and the availability of tissue for biomarker studies. The local ethics committee approved this study and eligible patients were acquired. Surgical procedures and systemic therapy were given at the discretion of the treating physician with or without targeted therapy, namely trastuzumab for human epidermal growth factor 2 (HER2^+^) BC patients. The medical charts of these patients were reviewed and their clinical details were included in a database.

We used a combination of immunohistochemical (IHC) markers for the classification of BC patients based on the pattern of expression of hormonal receptors (HR) and HER2 that identified three major distinct molecular BC subtypes: luminal tumors, which are HR positive and HER2^−^, HER2 amplified tumors and those tumors with no expression of any of the three receptors [30,31]. Tumor specimens from archival tumor biopsies of each patient were obtained and analyzed for different markers.

### CTC enrichment

Patients donated peripheral blood at the time of initial diagnosis. Control blood samples were drawn from 16 healthy volunteers with no history of malignant disease. 30 ml of blood was collected from each donor in three different Cell Save Preservatives blood collection Tubes (Veridex, LLC, Johnson & Johnson Company). Blood samples were maintained at room temperature and processed according to the protocols we have previously established [32]. For CTCs enrichment, we used the Carcinoma Cell Enrichment and Detection kit, MACS technology (Miltenyi Biotec), using magnetic beads labeled with a multi-CK-specific antibody (CK3-11D5) that recognizes CK 7, 8, 18 and 19. Patients were considered CTCs-positive if ≥1 CTC per 10 ml blood was detected. Samples negative for CK after selective immunomagnetic cell separation were considered CK-negative patients.

### Cell cultures and immunocytochemistry assay feasibility

BC cell lines were obtained from the European Collection of Cell Cultures (ECACC, Salisbury, UK). In the recovery experiments, we analyzed control samples with low numbers (10, 5, 1 cells) from MCF-7 (ECACC), SKBR3 (ECACC) and T47D (ECACC) human BC cell lines. Cells were spiked in 10 ml of venous blood from healthy volunteers and control experiments were performed at least in triplicate. Cytospins were prepared afterward by density gradient centrifugation and by immunomagnetic selection in the same way that the patient samples. Recovery rates of tumor cells spiked into normal blood at the low level control numbers were in the range of 40-60%. As negative controls, 16 blood samples from healthy volunteers without evidence of an epithelial malignancy were examined. Peripheral blood was drawn from the middle of vein puncture after the first 3 ml of blood were discarded. This precaution was undertaken in order to avoid contamination of the sample with epithelial cells from the skin during sample collection and to assure a high specificity of the method. We next tested technical feasibility determining protein expression by immunofluorescence (IF) and DNA amplification by FISH in isolated CTCs. We evaluated the range of expression seen in CTCs by presence or absence of staining using an anti-CK and EGFR antibody. Moreover, CD133 (AC133, Isotype: mouse IgG) (MiltenyiBiotec) expression in CTCs was analyzed by IF. TOP2A and HER2 amplification by FISH were determined in isolated CTCs. In the same way, CTCs negatives to CK expression were evaluated to EGFR, TOP2A/HER2 and CD133 expression, the methodology employed is shown in supplementary Fig. 1S.

HUVEC cell line was used as positive control for VIM and Slug expression. We evaluated their expression by presence or absence of staining using anti-VIM and anti-Slug antibodies (Santa Cruz Biotechnology). SKBR3 tumor cell line was used as negative control for both markers.

### CK and EGFR expression in CTCs

EGFR-positive cells were identified by immunofluorescence assays. The presence of CK cells was revealed by incubation with a freshly prepared Fast Red TR/Naphthol AS-MX substrate solution and identified under a microscope. EGFR^+^ cells were revealed by incubation with primary monoclonal anti-human EGFR (Dako) (dilution 1:25), followed by incubation with Alexa Flour 350 (Molecular Probes, Invitrogen). Epithelial tumor cells were identified and enumerated based on their red staining for CK-positive cells and blue staining for EGFR^+^ cells. The SKBR3 tumor cell line was used as positive control for EGFR. Identification and counting were done with a computerized fluorescence microscope Zeiss AXIO Imager.

### Vimentin and Slug expression in CTCs

The subpopulation of patients with CTCs negative for CK expression was selected by the immunomagnetic multi-CK enrichment method. The slides, negative for CK expression, were then stained with conjugated VIM-FITC (mouse monoclonal antibody) and Slug (rabbit polyclonal antibody) visualized with anti-rabbit Alexa Fluor 633 (Molecular Probes). Finally, CD45 (mouse monoclonal antibody, Santa Cruz Biotech) expression was revealed by incubation with an Alexa 405, to detect hematopoietic cells. Cells VIM^+^ and/or Slug+, CD45^−^were considered EMT-positive CTCs. After the identification of these markers, nuclear staining with DAPI was performed. Identification and counting were done with a computerized fluorescence microscope Zeiss AXIO Imager.

### EMT induction by TGFβ1 and/or EGF

2 × 10^5^ MCF-7 cells were seeded a day prior to starting the treatment at ~30-40% confluence in 6 well plates, then stimulated with recombinant human TGFβ_1_ (Santa Cruz Biotechnology) and/or EGF (Abcam, Cambridge) in DMEM without FBS medium at 0, 5 and 10 ng/ml or 0 and 20 ng/ml, respectively.

### Western blotting analysis

Protein extraction and Western blot analysis were performed in MCF-7 cells induced or non-induced with TGFβ1 and EGF. MCF-7 cells (5 × 10^5^) were plated in 6 wells plate followed by incubation with TGFβ1 and/or EGF at several concentrations (0, 5 and 10 ng/ml for TGFβ1 and 0 or 20ng/ml for EGF). After 72 h of treatment cells were lysed in sample buffer (62.76 mMTris– HCl pH 6.8, 5% 2-mercaptoethanol, 2% SDS, 10% glycerol, 0.5%bromophenol blue, and 100 mM dithiothreitol). Proteins (30 μg) were separated by SDS-PAGE (10%) in a Mini Protean II cell (Bio-Rad, Hercules, CA) and were transferred to a nitrocellulose membrane (80 V at room temperature for 30 min). Blots were treated with blocking solution (PBS TWIN 0.5% non-fat milk) for 1 h at room temperature and then reacted with primary antibody against VIM, Slug, Bcl-2, caspase 9, multi-CK, TGFβ R2 (Abcam, Cambridge) and EGFR (Santa Cruz Biotechnology, CA) at 1:1000 dilution overnight at 4°C. Then, membranes were washed and reincubated for 1h with a horseradish peroxidase-conjugated anti-IgG (Abcam, Cambridge) diluted at 1:1000. Protein–antibody complexes were visualized by enhanced chemiluminescence (ECL Prime western blotting Detection Reagent, GE Healthcare, Buckinghamshire) and using a densitrometric analysis system (J1·47t image processing system, National Institute of Health, USA).

### Caspase-3, -7 and -8 activities

Caspase-3, -7 and -8 activities were measured using Caspase-Glo^®^ 3/7 and Caspase-Glo^®^ 8 Assay kits (Promega, Madison, WI, USA) according to manufacturer's instructions. Briefly, MCF-7 cells were seeded at 5×10^4^ cells/well in 96-well, white-walled plates and treated with TGFβ1 and/or EGF, after cells were grown to 50% confluence. After incubation for 72 hours an equal volume of Caspase-Glo^®^ reagent was added. The plates were shaken at 500 rpm for 30 sec, incubated for 1 h, and the luminescence that is proportional to caspase 3/7 and 8 activities was determined by luminometer. Data are presented as the mean ± SD from three replicates.

### Confocal microscopy

Confocal images were obtained using a Zeiss LSM 710 confocal/multi photon laser scanning microscope equipped with Argon/2 laser (458, 477, 488, 514 nm) and a Titanium Sapphire laser (750 nm). The cells were viewed with several apochromatic objectives and images of different fields were taken. The microscope was set up to take multichannel images and the excitation and emission filter sets configured individually so that there is no fluorescence bleed-through between the channels. The argon (488 nm) laser with appropriate, emission filters was used for the visualization of Alexa Fluor 488. Alexa Fluor 488 was utilized to visualize EGFR, VIM and CK and an Alexa Fluor 633 was used to Slug. Adherent monolayer cultures of 1 × 10^4^ MCF7 cells per well were grown on glass coverslips for 72 hours with TGFβ1 (10 ng/ml) and with TGFβ1/EGF (10 ng/ml and 20 ng/ml, respectively) combination on culture slide (Becton Dickinson). Then, cells were washed and fixed with 3·7% formaldehyde in Dulbecco's PBS followed by permeabilization in 0·5% NP40. The cells were washed, blocked with 5% BSA in PBS, and incubated with primary antibody for overnight at 4°C and with Alexa Fluor 488 or Alexa Fluor 633-conjugated secondary antibody for one hour. The slides were then washed with PBS for 15 min air dried, and mounted with Vectashield^®^ mounting medium with 4′,6-diamidino-2-phenylindole (DAPI) (Cat# H-1200, Vector Laboratories). Images of three randomly-selected microscope fields of each sample were taken. The images were captured using a spinning objective confocal microscope at X60 magnification.

### RNA isolation and real-time PCR analysis

Real-time PCR was performed to assess the effect in the gene expression of TGFβ1 and/or EGF treatment on *EGFR*, *CK8*, *VIM* and *Slug*. MCF-7 cells (5 × 10^5^) were plated in 6 wells plate followed by incubation with TGFβ1 and/or EGF at several concentrations (0 and 10 ng/ml for TGFβ1 and 0 or 20ng/ml for EGF) for 72 h. Total cellular RNA was isolated using QIAcube equipment (QIAGEN) in conjunction with the miRNeasy Mini Kit (QIAGEN). The QuantiTect Reverse Transcription Kit (QIAGEN) was used for cDNA synthesis. Real-time PCR was performed using the KAPA SYBR Fast qPCR Kit (KAPABIOSYSTEMS) in conjunction with an Eco Illumina System (Illumina). The expression levels were normalized to corresponding for GAPDH values and are shown as fold change relative to the value of the control sample. All the analyses were carried out in triplicate. Primer sequences are reported in Table 1S.

### Wound healing assay

Migration of MCF-7 cells treated with TGFβ_1_and/or EGF for 72 h was measured using the *in vitro* wound-healing assay. Cells were seeded into 6-well plates and grown to 80% confluence. Wounds were created by scraping monolayer cells with a 200 μl pipette tip and non-adherent cells washed off with medium. At 0, 24 and 48 after the creation of wounds, cells were observed with a 10 X objective in an Axiovert 40 CFC Zeiss (Carl Zeiss meditec group, Germany) photomicroscope. Images were acquired with an AxioCam ICc3 Zeiss (Carl Zeiss meditec group, Germany) color digital camera. Wound distances were measured at each time point and presented in pixels. J·147t Software was used to quantify the wound area. All experiments were plated in triplicate wells and were carried out at least three times

### Statistical analysis

We used χ^2^ or Student's *t*-test to assess the correlation between VIM and Slug expression in CTCs CK^−^samples of non-metastatic BC with clinical outcomes and EGFR expression. The presence of at least 1 CTC per 10 ml was considered a positive result, according to the reported analytic detection limit of our assay (32). We evaluated the range of expression of VIM and Slug seen in CK-negative CTCs by presence or absence of staining. SPSS 14.0 software was used for statistical analysis. Data is presented as means or numbers (%). Two-tailed *p*<0.05 values were considered statistically significant.

## SUPPLEMENTARY FIGURES AND TABLE


