# Supplementary material for: EMT and EGFR in CTCs cytokeratin negative non-metastatic breast cancer
Source: Oncotarget. 2014 Jul 14;5(17):7486–97. doi: 10.18632/oncotarget.2217 (PMC4202138; doi:10.18632/oncotarget.2217)
Supplement: Supplementary file 1 [file oncotarget-05-7486-s001.pdf]

EMT and EGFR in CTCs cytokeratin negative non-metastatic breast cancer

Supplementary Material

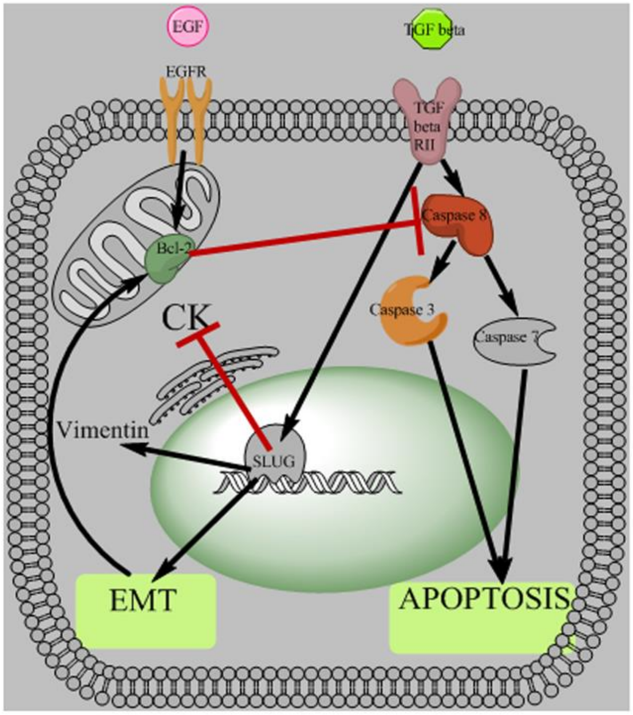

Figure 1S: Schema of the methodology used to assess the characterization by IF of CK negative samples. Patients donated three 10 ml samples of peripheral blood at the time of initial diagnosis. Equal samples were obtained from healthy volunteers. In each tube CTCs negatives to CK expression were further evaluated for VIM/Slug expression and for EGFR (A), TOP2A/HER2 (B) and CD133 (C) expression, respectively.

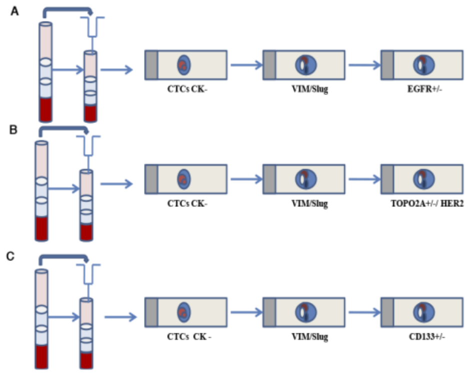

Figure 2S: Schematic representation of the mechanisms by which TGFβ1 and EGFR activation may enhance EMT phenomenon in CTCs negative for CK in BC patients

Table 1S: Primer sequences for RT-qPCR

Gene	Primer Sequence	
VIMENTIN	Forward	5′ GTTTCCCCTAAACCGCTAGG 3′
	Reverse	5′ AGCGAGAGTGGCAGAGGA 3′
SLUG	Forward	5´ ′TGGTTGCTTCAAGGACACAT 3′
	Reverse	5′ GTTGCAGTGAGGGCAAGAA 3′
CK8	Forward	5′ GAATGAATGGGGTGAGCTGG 3′
	Reverse	5′ CCTGATGGACATGGTAGAGGC 3′
EGFR	Forward	5´ TGCACCTACGGATGCACTG 3´
	Reverse	5´ CGATGGACGGGATCTTAGGC 3´
GAPDH	Forward	5′ GGACTCATGACCACAGTCCATGCC 3′
	Reverse	5′ TCAGGGATGACCTTGCCCACAG 3′
